# Analysis of COVID-19 epidemic model with sumudu transform

**DOI:** 10.3934/publichealth.2022022

**Published:** 2022-02-14

**Authors:** Muhammad Farman, Muhammad Azeem, M. O. Ahmad

**Affiliations:** Department of Mathematics and Statistics, The University of Lahore, Lahore, Pakistan

**Keywords:** epidemic model, stability, unique solution, sumudu transform, fractional derivative

## Abstract

In this paper, we develop a time-fractional order COVID-19 model with effects of disease during quarantine which consists of the system of fractional differential equations. Fractional order COVID-19 model is investigated with ABC technique using sumudu transform. Also, the deterministic mathematical model for the quarantine effect is investigated with different fractional parameters. The existence and uniqueness of the fractional-order model are derived using fixed point theory. The sumudu transform can keep the unity of the function, the parity of the function, and has many other properties that are more valuable. Solutions are derived to investigate the influence of fractional operator which shows the impact of the disease during quarantine on society.

## 1. Introduction

A mathematical model is a helpful tool to recognize the conduct of an infection when it starts to affect the community and it is useful to analyze under what conditions it can be screened out or to be continued [1]. A virus is known as infectious when any disease is transferred from one person to another via different ways of transmission like droplets generated when an infected person coughs, sneezes, or exhales, or direct contact with another human, water, or any physical product. To analyze this type of transmission we need some authentic mathematical tools in which few of them are difference equations, initial conditions, working parameters, and statistical estimation. In this new era, new mathematical techniques give us more updated and reliable tools to understand many diseases or infections in epidemiology and even give us updated strategies to control disease or infection in different and suitable conditions [2].

From all of the viruses, the COVID-19 is gradually becoming a watershed pandemic in the antiquity of the planet. COVID-19 is an abbreviation of Coronavirus disease which started in 2019. In December of 2019, the first case of COVID-19 was observed in Wuhan, the city of China [3]. The common symptoms of COVID-19 are loss of smell and taste, fever, dry cough, shortening of breath, fatigue, muscle, and joint pain, phlegm production, sore throat, headache, and chills, these symptoms vary from person to person. The most common incubation period ranges from 1 to 12 days. COVID-19 spreads by physical interaction between individuals. Use of masks, sanitizer, and having a distance of 2 m between individuals results in minimizing the spread of the virus up to much extent [4]. These vaccines played a bold role in minimizing the spread of COVID-19. The main focused area of this spread is the working area, schools, offices, markets, and other open circles [5],[6].

Fractional derivative was originated in 1695. If we describe the list of fractional derivatives then it is divided into two types. Caputo, Riemann-Liouville, and Katugampola [7] are fractional derivatives with the singular kernel. Caputo-Fabrizio(exponential) [8] and ABC(Mittag-Leffler) [9] are fractional derivatives without singular kernels. Fractional calculus has very vast application properties in our daily life. It is being used in chemical, biological, physical, finance, pharmaceutical, engineering [10],[11], and many other fields [12]–[14]. FFD is mostly used because it gives a realistic way of representation of our model and hence we have used this same for representing our COVID-19 epidemics [15]–[18]. A time-fractional compartmental model for the COVID-19 pandemic [21] and classical SIR model for COVID-19 in United States is study in [22]. The COVID-19 pandemic (caused by SARS-CoV-2) has introduced significant challenges for accurate prediction of population morbidity and mortality by traditional variable-based methods of estimation. Challenges to modeling include inadequate viral physiology comprehension and fluctuating definitions of positivity between national-to-international data. This paper proposes that accurate forecasting of COVID-19 caseload may be best preformed non-perimetrically, by vector autoregressive (VAR) of verifiable data regionally [23]. Fundamental properties of the new generalized fractional derivatives in the sense of Caputo and RiemannLiouville are rigorously studied and its related work [24]–[26]. COVID-19 Decision-Making System (CDMS) was developed to study disease transmission in [27]. The change in atmospheric pollution from a public lockdown in Greece introduced to curb the spread of the COVID-19 is examined based on ground-based and satellite observations and some related issues in [28]–[30].

## 2. Basic concepts

The fractional-order derivative of AB in Reimann Liouville-Caputo sense (ABC) [19] is given by

$${}_{\gamma }^{ABC}D_{t}^{\gamma }\{f(t)\}=\frac{AB(\gamma )}{m-\gamma }\int_{\gamma }^{t}\frac{d^{m}}{dw^{m}}f(w)E_{\gamma }[-\gamma \frac{{\left(t-w\right)}^{\gamma }}{m-\gamma }]dw,m-1<\gamma <m$$ (1)

where *E_γ_* is the Mittag-Leffler function and *AB*(*γ*) is a normalization function and *AB*(0) = *AB*(1) = 1. The Laplace transform of above is given by

$${[}_{\gamma }^{ABC}D_{t}^{\gamma }f(t)](s)=\frac{AB(\gamma )}{1-\gamma }\frac{s^{\gamma }L[f(t)](s)-s^{\gamma -1}f(0)}{s^{\gamma }+\frac{\gamma }{1-\gamma }}$$ (2)

with the aid of sumudu transformation, we get

$$ST{[}_{\gamma }^{ABC}D_{t}^{\gamma }f(t)](s)=\frac{B(\gamma )}{1-\gamma }(\gamma \Gamma (\gamma +1)E_{\gamma }(-\frac{1}{1-\gamma }\nu^{\gamma }))\times [ST(f(t))-f(0)]$$ (3)

The ABC fractional integral of order *γ* of a function *f*(*t*) is given by

$${}_{\gamma }^{ABC}I_{t}^{\gamma }\{f(t)\}=\frac{1-(\gamma )}{B-\gamma }f(t)+\frac{\left(\gamma \right)}{B(\gamma )\Gamma (\gamma )}\int_{\gamma }^{t}f(s)(t-s{)}^{\gamma -1}ds$$ (4)

## 3. Materials and methods

In this section, consider the improved SEIR model given in [20] having compartments SEIQRPD, where S represents the number of uninfected individuals, E represents infected individuals at the time *t* but still in incubation period (without clinical symptoms and low infectivity), I represents the number of infected individuals at the time *t* (with obvious clinical symptoms), *Q* represents the number of individuals who have been diagnosed and isolated at the time *t*, *R* represents the number of recovered individuals at the time *t*, *P* represents the number of susceptible individuals who are not exposed to the external environment at the time *t* and *D* represents the number of death cases at time *t*.

$$\begin{array}{c} {}_{0}^{ABC}D_{t}^{\gamma }S(t)=-\beta_{1}(t)a_{1}(S(t))b_{1}(I(t))-\beta_{2}a_{2}(S(t))b_{2}(E(t))-\rho S(t),\\ {}_{0}^{ABC}D_{t}^{\gamma }E(t)=\beta_{1}(t)a_{1}(S(t))b_{1}(I(t))+\beta_{2}a_{2}(S(t))b_{2}(E(t))-\varepsilon E(t),\\ {}_{0}^{ABC}D_{t}^{\gamma }I(t)=\varepsilon E(t)-\delta I(t),\\ {}_{0}^{ABC}D_{t}^{\gamma }Q(t)=\delta I(t)-(\lambda (t)+\kappa (t))Q(t),\\ {}_{0}^{ABC}D_{t}^{\gamma }R(t)=\lambda (t)Q(t),\\ {}_{0}^{ABC}D_{t}^{\gamma }P(t)=\rho S(t)\\ {}_{0}^{ABC}D_{t}^{\gamma }D(t)=\kappa (t)Q(t) \end{array}$$ (5)

here $\beta_{1}(t)=\sigma_{1}exp(-\sigma_{2}t),\lambda (t)=\lambda_{1}(1-exp(\lambda_{2}t))$ and $\kappa (t)=\kappa_{1}exp(-\kappa_{2}t)$ $\sigma_{1},\sigma_{2},\lambda_{1},\lambda_{2},\kappa_{1}$ and *κ*_2_ are the parameters which are all positive, here simulation is used by the *σ* affect of government control. It should be emphasized that the protection rate *ρ* for susceptible individuals also reflects the intensity of government control [18]. ${}_{0}^{ABC}D_{t}^{\gamma }$, is the ABC sense fractional derivative with 0<γ≤1. The initial conditions of the system Eq 5 are:

$$\begin{array}{c} S_{0}(t)=S(0),E_{0}(t)=E(0),I_{0}(t)=I(0),Q_{0}(t)=Q(0)\\ R_{0}(t)=R(0),P_{0}(t)=P(0),D_{0}(t)=D(0) \end{array}$$ (6)

applying ST operator on both sides, we get

$$\begin{array}{c} O_{\gamma }E_{\gamma }(-\frac{1}{1-\gamma }\omega^{\gamma })[ST(S(t))-S(0)]\\ =ST[-\beta_{1}(t)a_{1}(S(t))b_{1}(I(t))-\beta_{2}a_{2}(S(t))b_{2}(E(t))-\rho S(t)],\\ O_{\gamma }E_{\gamma }(-\frac{1}{1-\gamma }\omega^{\gamma })[ST(E(t))-E(0)]\\ =ST[\beta_{1}(t)a_{1}(S(t))b_{1}(I(t))+\beta_{2}a_{2}(S(t))b_{2}(E(t))-\varepsilon E(t)],\\ O_{\gamma }E_{\gamma }(-\frac{1}{1-\gamma }\omega^{\gamma })[ST(I(t))-I(0)]\\ =ST[\varepsilon E(t)-\delta I(t)],\\ O_{\gamma }E_{\gamma }(-\frac{1}{1-\gamma }\omega^{\gamma })[ST(Q(t))-Q(0)]\\ =ST[\delta I(t)-(\lambda (t)+\kappa (t))Q(t)],\\ O_{\gamma }E_{\gamma }(-\frac{1}{1-\gamma }\omega^{\gamma })[ST(R(t))-R(0)]\\ =ST[\lambda (t)Q(t)],\\ O_{\gamma }E_{\gamma }(-\frac{1}{1-\gamma }\omega^{\gamma })[ST(P(t))-P(0)]\\ =ST[\rho S(t)],\\ O_{\gamma }E_{\gamma }(-\frac{1}{1-\gamma }\omega^{\gamma })[ST(D(t))-D(0)]\\ =ST[\kappa (t)Q(t)] \end{array}$$ (7)

where $O_{\gamma }=\frac{B(\gamma )\gamma \Gamma (\gamma +1)}{1-\gamma }$ system Eq 7 becomes

$$\begin{array}{c} ST[S(t)]=S(0)+\frac{1}{O_{\gamma }E_{\gamma }(-\frac{1}{1-\gamma }\omega^{\gamma })}\\ \times ST[-\beta_{1}(t)a_{1}(S(t))b_{1}(I(t))-\beta_{2}a_{2}(S(t))b_{2}(E(t))-\rho S(t)],\\ ST[E(t)]=E(0)+\frac{1}{O_{\gamma }E_{\gamma }(-\frac{1}{1-\gamma }\omega^{\gamma })}\\ \times ST[\beta_{1}(t)a_{1}(S(t))b_{1}(I(t))+\beta_{2}a_{2}(S(t))b_{2}(E(t))-\varepsilon E(t)],\\ ST[I(t)]=I(0)+\frac{1}{O_{\gamma }E_{\gamma }(-\frac{1}{1-\gamma }\omega^{\gamma })}\\ \times ST[\varepsilon E(t)-\delta I(t)],\\ ST[Q(t)]=Q(0)+\frac{1}{O_{\gamma }E_{\gamma }(-\frac{1}{1-\gamma }\omega^{\gamma })}\\ \times ST[\delta I(t)-(\lambda (t)+\kappa (t))Q(t)],\\ ST[R(t)]=R(0)+\frac{1}{O_{\gamma }E_{\gamma }(-\frac{1}{1-\gamma }\omega^{\gamma })}\\ \times ST[\lambda (t)Q(t)],\\ ST[P(t)]=P(0)+\frac{1}{O_{\gamma }E_{\gamma }(-\frac{1}{1-\gamma }\omega^{\gamma })}\\ \times ST[\rho S(t)],\\ ST[D(t)]=D(0)+\frac{1}{O_{\gamma }E_{\gamma }(-\frac{1}{1-\gamma }\omega^{\gamma })}\\ \times ST[\kappa (t)Q(t)] \end{array}$$ (8)

taking inverse Sumudu Transform on both sides, we get

$$\begin{array}{c} S(t)=S(0)+ST^{-1}\{\frac{1}{O_{\gamma }E_{\gamma }(-\frac{1}{1-\gamma }\omega^{\gamma })}\\ \times ST[-\beta_{1}(t)a_{1}(S(t))b_{1}(I(t))-\beta_{2}a_{2}(S(t))b_{2}(E(t))-\rho S(t)]\},\\ E(t)=E(0)+ST^{-1}\{\frac{1}{O_{\gamma }E_{\gamma }(-\frac{1}{1-\gamma }\omega^{\gamma })}\\ \times ST[\beta_{1}(t)a_{1}(S(t))b_{1}(I(t))+\beta_{2}a_{2}(S(t))b_{2}(E(t))-\varepsilon E(t)]\},\\ I(t)=I(0)+ST^{-1}\{\frac{1}{O_{\gamma }E_{\gamma }(-\frac{1}{1-\gamma }\omega^{\gamma })}\\ \times ST[\varepsilon E(t)-\delta I(t)]\},\\ Q(t)=Q(0)+ST^{-1}\{\frac{1}{O_{\gamma }E_{\gamma }(-\frac{1}{1-\gamma }\omega^{\gamma })}\\ \times ST[\delta I(t)-(\lambda (t)+\kappa (t))Q(t)]\},\\ R(t)=R(0)+ST^{-1}\{\frac{1}{O_{\gamma }E_{\gamma }(-\frac{1}{1-\gamma }\omega^{\gamma })}\\ \times ST[\lambda (t)Q(t)]\},\\ P(t)=P(0)+ST^{-1}\{\frac{1}{O_{\gamma }E_{\gamma }(-\frac{1}{1-\gamma }\omega^{\gamma })}\\ \times ST[\rho S(t)]\},\\ D(t)=D(0)+ST^{-1}\{\frac{1}{O_{\gamma }E_{\gamma }(-\frac{1}{1-\gamma }\omega^{\gamma })}\\ \times ST[\kappa (t)Q(t)]\} \end{array}$$ (9)

Therefore, the following is obtained

$$\begin{array}{c} S_{\left(m+1\right)}(t)=S_{m}(0)+ST^{-1}\{\frac{1}{O_{\gamma }E_{\gamma }(-\frac{1}{1-\gamma }\omega^{\gamma })}\\ \times ST[-\beta_{1}a_{1}(S_{m}(t))b_{1}(I_{m}(t))-\beta_{2}a_{2}(S_{m}(t))b_{2}(E_{m}(t))-\rho S_{m}(t)]\},\\ E_{\left(m+1\right)}(t)=E_{m}(0)+ST^{-1}\{\frac{1}{O_{\gamma }E_{\gamma }(-\frac{1}{1-\gamma }\omega^{\gamma })}\\ \times ST[\beta_{1}a_{1}(S_{m}(t))b_{1}(I_{m}(t))+\beta_{2}a_{2}(S_{m}(t))b_{2}(E_{m}(t))-\varepsilon E_{m}(t)]\},\\ I_{\left(m+1\right)}(t)=I_{m}(0)+ST^{-1}\{\frac{1}{O_{\gamma }E_{\gamma }(-\frac{1}{1-\gamma }\omega^{\gamma })}\\ \times ST[\varepsilon E_{m}(t)-\delta I_{m}(t)]\},\\ Q_{\left(m+1\right)}(t)=Q_{m}(0)+ST^{-1}\{\frac{1}{O_{\gamma }E_{\gamma }(-\frac{1}{1-\gamma }\omega^{\gamma })}\\ \times ST[\delta I_{m}(t)-(\lambda +\kappa )Q_{m}(t)]\},\\ R_{\left(m+1\right)}(t)=R_{m}(0)+ST^{-1}\{\frac{1}{O_{\gamma }E_{\gamma }(-\frac{1}{1-\gamma }\omega^{\gamma })}\\ \times ST[\lambda Q_{m}(t)]\},\\ P_{\left(m+1\right)}(t)=P_{m}(0)+ST^{-1}\{\frac{1}{O_{\gamma }E_{\gamma }(-\frac{1}{1-\gamma }\omega^{\gamma })}\\ \times ST[\rho S_{m}(t)]\},\\ D_{\left(m+1\right)}(t)=D_{m}(0)+ST^{-1}\{\frac{1}{O_{\gamma }E_{\gamma }(-\frac{1}{1-\gamma }\omega^{\gamma })}\\ \times ST[\kappa Q_{m}(t)]\} \end{array}$$ (10)

Let's consider Eq 10, and then we get

$$\begin{array}{l} S(t)=\underset{m\to \infty }{lim}S_{m}(t);E(t)=\underset{m\to \infty }{lim}E_{m}(t);I(t)=\underset{m\to \infty }{lim}I_{m}(t);\\ Q(t)=\underset{m\to \infty }{lim}Q_{m}(t);R(t)=\underset{m\to \infty }{lim}R_{m}(t);P(t)=\underset{m\to \infty }{lim}P_{m}(t));D(t)=\underset{m\to \infty }{lim}D_{m}(t) \end{array}$$ (11)

**Theorem 3.1:** Let (X,|.|) be a Banach space and H a self-map of X satisfying

$$∥H_{r}-H_{x}∥\leq \theta ∥X-H_{r}∥+\theta ∥r-x∥$$ (12)

for all r,x∈X, where 0≤θ<1. Assume that H is Pichard H-stable

Let us consider Eq 10, and we obtain

$$\frac{1}{O_{\gamma }E_{\gamma }(-\frac{1}{1-\gamma }\omega^{\gamma })}$$ (13)

the above equation is associated with the fractional Lagrange multiplier.

**Proof**

Define K be a self-map is given by

$$\begin{array}{c} K[S_{\left(m+1\right)}(t)]=S_{\left(m+1\right)}(t)=S_{m}(0)+ST^{-1}[\frac{1}{O_{\gamma }E_{\gamma }(-\frac{1}{1-\gamma }\omega^{\gamma })}\\ \times ST[-\beta_{1}a_{1}(S_{m}(t))b_{1}(I_{m}(t))-\beta_{2}a_{2}(S_{m}(t))b_{2}(E_{m}(t))-\rho S_{m}(t)]\\ K[E_{\left(m+1\right)}(t)]=E_{\left(m+1\right)}(t)=E_{m}(0)+ST^{-1}[\frac{1}{O_{\gamma }E_{\gamma }(-\frac{1}{1-\gamma }\omega^{\gamma })}\\ \times ST[\beta_{1}a_{1}(S_{m}(t))b_{1}(I_{m}(t))+\beta_{2}a_{2}(S_{m}(t))b_{2}(E_{m}(t))-\varepsilon E_{m}(t)]\\ K[I_{\left(m+1\right)}(t)]=I_{\left(m+1\right)}(t)=I_{m}(0)+ST^{-1}[\frac{1-}{O_{\gamma }E_{\gamma }(-\frac{1}{1-\gamma }\omega^{\gamma })}\\ \times ST[\varepsilon E_{m}(t)-\delta I_{m}(t)]\\ K[Q_{\left(m+1\right)}(t)]=Q_{\left(m+1\right)}(t)=Q_{m}(0)+ST^{-1}[\frac{1}{O_{\gamma }E_{\gamma }(-\frac{1}{1-\gamma }\omega^{\gamma })}\\ \times ST[\delta I_{m}(t)-(\lambda +\kappa )Q_{m}(t)]\\ K[R_{\left(m+1\right)}(t)]=R_{\left(m+1\right)}(t)=R_{m}(0)+ST^{-1}[\frac{1}{O_{\gamma }E_{\gamma }(-\frac{1}{1-\gamma }\omega^{\gamma })}\\ \times ST[\lambda Q_{m}(t)]\\ K[P_{\left(m+1\right)}(t)]=P_{\left(m+1\right)}(t)=P_{m}(0)+ST^{-1}[\frac{1}{O_{\gamma }E_{\gamma }(-\frac{1}{1-\gamma }\omega^{\gamma })}\\ \times ST[\rho S_{m}(t)]\\ K[D_{\left(m+1\right)}(t)]=D_{\left(m+1\right)}(t)=D_{m}(0)+ST^{-1}[\frac{1}{O_{\gamma }E_{\gamma }(-\frac{1}{1-\gamma }\omega^{\gamma })}\\ \times ST[\kappa Q_{m}(t)] \end{array}$$ (14)

Applying the properties of the norm and triangular inequality, we get

$$\begin{array}{c} ∥K[S_{m}(t)]-K[S_{n}(t)]∥\leq ∥S_{m}(t)-S_{n}(t)∥+∥ST^{-1}\{\frac{1}{O_{\gamma }E_{\gamma }(-\frac{1}{1-\gamma }\omega^{\gamma })}\\ \times ST[-\beta_{1}a_{1}S_{m}(t)b_{1}(I_{m}(t))-\beta_{2}a_{2}(S_{m}(t))b_{2}(E_{m}(t))-\rho S_{m}(t)]\}\\ -ST^{-1}\{\frac{1}{O_{\gamma }E_{\gamma }(-\frac{1}{1-\gamma }\omega^{\gamma })}\\ \times ST[-\beta_{1}a_{1}(S_{n}(t))b_{1}(I_{n}(t))-\beta_{2}a_{2}(S_{n}(t))b_{2}(E_{n}(t))-\rho S_{n}(t)]\}∥,\\ ∥K[E_{m}(t)]-K[E_{n}(t)]∥\leq ∥E_{m}(t)-E_{n}(t)∥+∥ST^{-1}[\frac{1}{O_{\gamma }E_{\gamma }(-\frac{1}{1-\gamma }\omega^{\gamma })}\\ \times ST[\beta_{1}a_{1}(S_{m}(t))b_{1}(I_{m}(t))+\beta_{2}a_{2}(S_{m}(t))b_{2}(E_{m}(t))-\varepsilon E_{m}(t)]\}\\ -ST^{-1}[\frac{1}{O_{\gamma }E_{\gamma }(-\frac{1}{1-\gamma }\omega^{\gamma })}\\ \times ST[\beta_{1}a_{1}(S_{n}(t))b_{1}(I_{n}(t))+\beta_{2}a_{2}(S_{n}(t))b_{2}(E_{n}(t))-\varepsilon E_{n}(t)]\}∥,\\ ∥K[I_{m}(t)]-K[I_{n}(t)]∥\leq ∥I_{m}(t)-I_{n}(t)∥+∥ST^{-1}[\frac{1}{O_{\gamma }E_{\gamma }(-\frac{1}{1-\gamma }\omega^{\gamma })}\\ \times ST[\varepsilon (E_{m}(t))-\delta (I_{m}(t)))]\}-ST^{-1}[\frac{1}{O_{\gamma }E_{\gamma }(-\frac{1}{1-\gamma }\omega^{\gamma })}\\ \times ST[\varepsilon E_{n}(t)-\delta I_{n}(t)]\}∥,\\ ∥K[Q_{m}(t)]-K[Q_{n}(t)]∥\leq ∥Q_{m}(t)-Q_{n}(t)∥+∥ST^{-1}[\frac{1}{O_{\gamma }E_{\gamma }(-\frac{1}{1-\gamma }\omega^{\gamma })}\\ \times ST[\delta I_{m}(t)-(\lambda +\kappa )Q_{m}(t))]\}-ST^{-1}[\frac{1}{O_{\gamma }E_{\gamma }(-\frac{1}{1-\gamma }\omega^{\gamma })}\\ \times ST[\delta I_{n}(t)-(\lambda +\kappa )Q_{n}(t)]\}∥\\ ∥K[R_{m}(t)]-K[R_{n}(t)]∥\leq ∥R_{m}(t)-R_{n}(t)∥+∥ST^{-1}[\frac{1}{O_{\gamma }E_{\gamma }(-\frac{1}{1-\gamma }\omega^{\gamma })}\\ \times ST[\lambda (Q_{m}(t))]\}-ST^{-1}[\frac{1}{O_{\gamma }E_{\gamma }(-\frac{1}{1-\gamma }\omega^{\gamma })}\\ \times ST[\lambda Q_{n}(t)]\}∥,\\ ∥K[P_{m}(t)]-K[P_{n}(t)]∥\leq ∥P_{m}(t)-P_{n}(t)∥+∥ST^{-1}[\frac{1}{O_{\gamma }E_{\gamma }(-\frac{1}{1-\gamma }\omega^{\gamma })}\\ \times ST[\rho S_{m}(t)]\}-ST^{-1}[\frac{1}{O_{\gamma }E_{\gamma }(-\frac{1}{1-\gamma }\omega^{\gamma })}\\ \times ST[\rho S_{n}(t)]\}∥,\\ ∥K[D_{m}(t)]-K[D_{n}(t)]∥\leq ∥D_{m}(t)-D_{n}(t)∥+∥ST^{-1}[\frac{1}{O_{\gamma }E_{\gamma }(-\frac{1}{1-\gamma }\omega^{\gamma })}\\ \times ST[\kappa Q_{m}(t)]\}-ST^{-1}[\frac{1}{O_{\gamma }E_{\gamma }(-\frac{1}{1-\gamma }\omega^{\gamma })}\\ \times ST[\kappa Q_{n}(t)]\}∥ \end{array}$$ (15)

K fulfills the conditions associated with theorem 3.1 when

$$\theta =(0,0,0,0,0,0,0),\theta =(\begin{array}{l} ∥S_{m}(t)-S_{n}(t)∥\times ∥-S_{m}(t)+S_{n}(t)∥\\ -∥\beta_{1}a_{1}(S_{m}(t)-S_{n}(t))b_{1}(I_{m}(t)-I_{n}(t))∥\\ -∥\beta_{2}a_{2}(S_{m}(t)-S_{n}(t))b_{2}(E_{m}(t)-E_{n}(t))∥\\ -∥\rho (S_{m}(t)-S_{n}(t))∥\\ \times ∥E_{m}(t)-E_{n}(t)∥\times ∥-E_{m}(t)+E_{n}(t)∥\\ +∥\beta_{1}a_{1}(S_{m}(t)-S_{n}(t))b_{2}(E_{m}(t)-E_{n}(t))∥\\ -∥\varepsilon (E_{m}(t)-E_{n}(t))∥\\ \times ∥I_{m}(t)-I_{n}(t)∥\times ∥-I_{m}(t)+I_{n}(t)∥\\ +∥\varepsilon (E_{m}(t)-E_{n}(t))∥-\delta ∥I_{m}(t)-I_{n}(t)∥\\ \times ∥Q_{m}(t)-Q_{n}(t)∥\times ∥-Q_{m}(t)+Q_{n}(t)∥\\ +∥\delta (I_{m}(t)-I_{n}(t))∥-(\lambda (t)+\kappa (t))∥Q_{m}(t)-Q_{n}(t)∥\\ \times ∥R_{m}(t)-R_{n}(t)∥\times ∥-R_{m}(t)+R_{n}(t)∥\\ +∥\lambda (Q_{m}(t)-Q_{n}(t))∥\\ \times ∥P_{m}(t)-P_{n}(t)∥-∥-P_{m}(t)+P_{n}(t)∥\\ +∥\rho (S_{m}(t)-S_{n}(t))∥\\ \times ∥D_{m}(t)-D_{n}(t)∥-∥-D_{m}(t)+D_{n}(t)∥\\ +∥\kappa (Q_{m}(t)-Q_{n}(t))∥ \end{array}$$ (16)

and we add that K is Picard K-stable.

## 4. Numerical results and discussion

**Figure 1.**
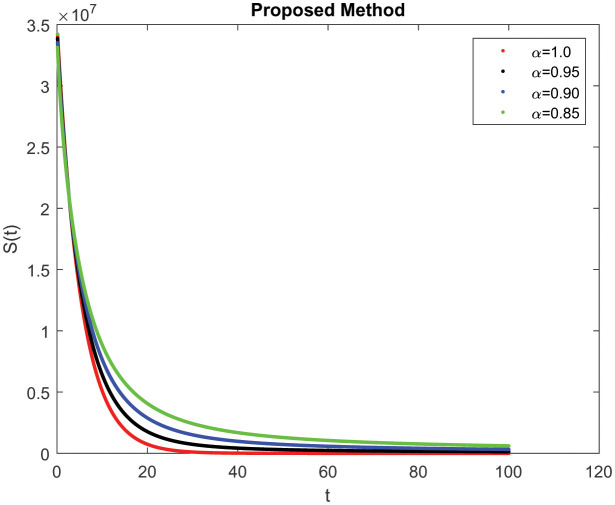
Simulation of *S*(*t*) at the time *t* with parametric value of *γ* with ABC.

**Figure 2.**
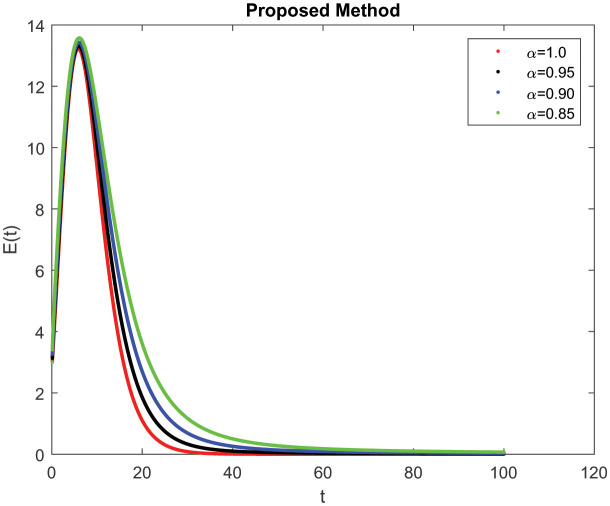
Simulation of *E*(*t*) at the time *t* with parametric value of *γ* with ABC.

**Figure 3.**
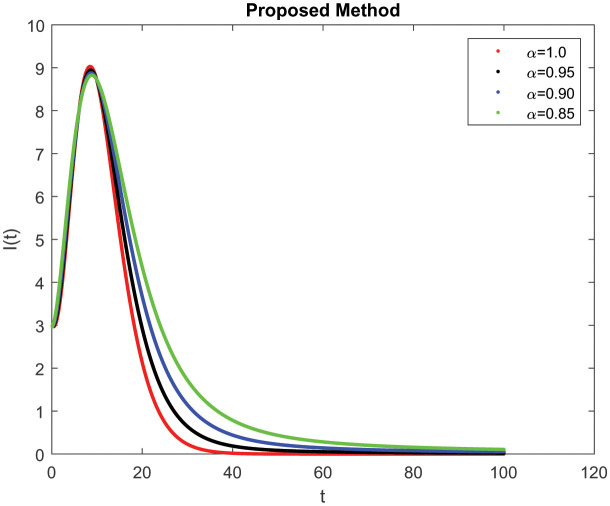
Simulation of *I*(*t*) at the time *t* with parametric value of *γ* with ABC.

**Figure 4.**
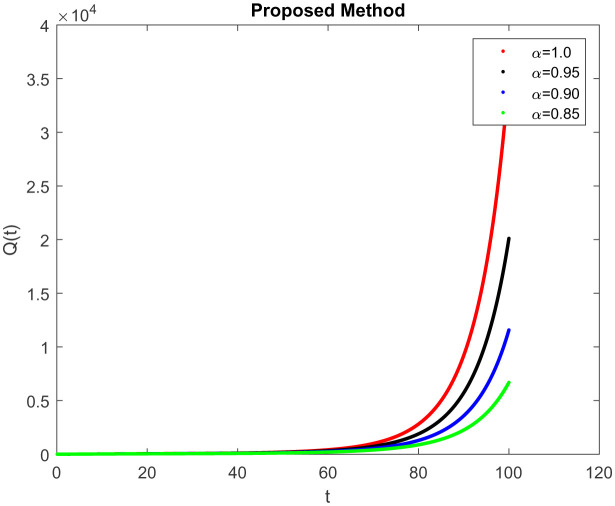
Simulation of *Q*(*t*) at the time *t* with parametric value of *γ* with ABC.

**Figure 5.**
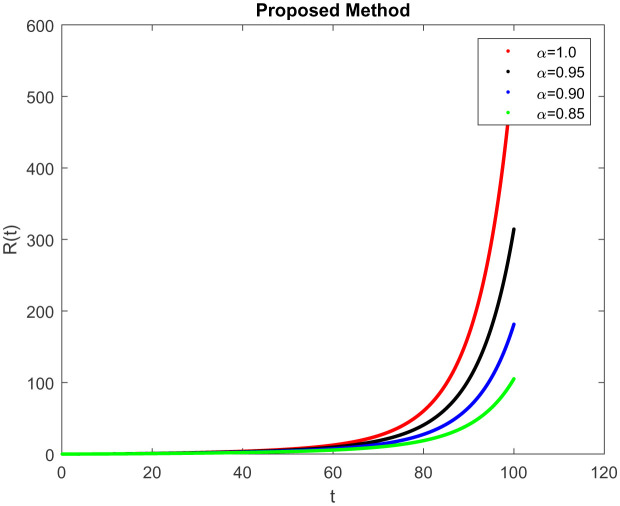
Simulation of *R*(*t*) at the time *t* with parametric value of *γ* with ABC.

**Figure 6.**
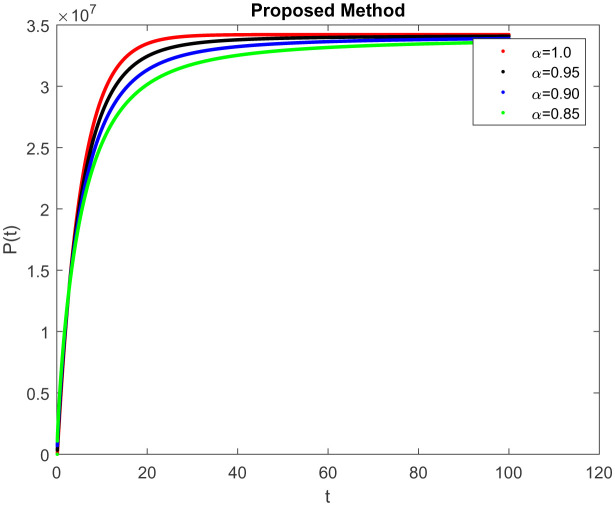
Simulation of *P*(*t*) at the time *t* with parametric value of *γ* with ABC.

**Figure 7.**
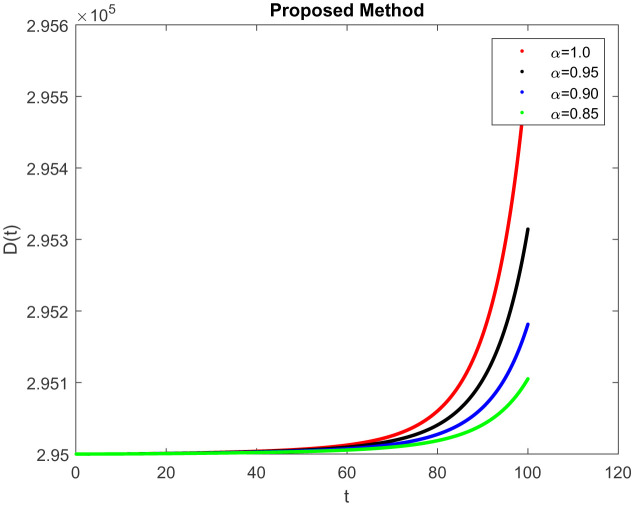
Simulation of *D*(*t*) at the time *t* with parametric value of *γ* with ABC.

In this section, consider the numerical simulations of the proposed scheme using the ABC technique for the fractional-order COVID-19 model. Figure 1 shows the simulation *S*(*t*) represents the number of uninfected individuals. Shows a deep decreasing curve till point (20, 0.25) and then becomes constant and reduced to zero at (100, 0). Figure 2 shows the simulation *E*(*t*) of infected individuals but still is in the incubation period (without clinical symptoms and low infectivity). Figure 3 *I*(*t*) which represents the number of infected individuals. Here the graph shows a rapid increase (10, 9) and then decrease rapidly with the same rate and then it becomes constant at (100, 0). Figure 4 represents the number of individuals who have been diagnosed and isolated. Figures 5 and 6 shows the simulation of recovered individuals and those not exposed to the external environment respectively. Figure 7 shows the simulation *D*(*t*), which represents the death due to increasing or decreasing the infection rate of COVID-19 in society. It can be easily observed from all figures the solution will converge to steady-state and lie in the bounded domain by decreasing the fractional value. Moreover, it has been demonstrated that physical processes are better well described using the derivatives of fractional order which are more accurate and reliable in comparison with the classical-order derivatives. Moreover, it can be seen from all figures that tell that all infected individual comes zero after a few days due to the quarantine effect. The behavior of the dynamics obtained for different instances of fractional-order was shown in the form of numerical results has been reported.

## 5. Conclusions

We consider the COVID-19 model with fractional operator for this work to check the dynamical behavior of infection of disease in society. In this regard, ABC derivative gave a realistic approach to analyze the effect of disseise during Quarantine which will be helpful for such type of epidemic. The existence and unique solution of the fractional-order model was made with the help of fixed point theory and iterative method. Numerical simulation has been made to check the actual behavior of the COVID-19 effect during quarantine which shows that infected individuals start decreasing after a few days. Such kind of results are very helpful for planning, decision-making, and developing control strategies to overcome the effect of COVID-19 in society.
